# What is the evidence for a role for diet and nutrition in osteoarthritis?

**DOI:** 10.1093/rheumatology/key011

**Published:** 2018-04-17

**Authors:** Sally Thomas, Heather Browne, Ali Mobasheri, Margaret P Rayman

**Affiliations:** 1 Department of Nutritional Sciences, School of Biosciences and Medicine, Faculty of Health and Medical Sciences, University of Surrey, Guildford, UK; 2 School of Veterinary Medicine, Faculty of Health and Medical Sciences, University of Surrey, Guildford, UK; 3 Arthritis Research UK Centre for Sport, Exercise and Osteoarthritis, Arthritis Research UK Centre for Musculoskeletal Ageing Research, Queen’s Medical Centre, Nottingham, UK; 4 Department of Regenerative Medicine, State Research Institute, Centre for Innovative Medicine, Santariskiu 5, 08661 Vilnius, Republic of Lithuania

**Keywords:** nutrition, osteoarthritis (OA), obesity, diabetes, metabolic syndrome, polyunsaturated fatty acids (PUFAs), cholesterol, anti-oxidants, vitamin D, vitamin K

## Abstract

As current treatment options in OA are very limited, OA patients would benefit greatly from some ability to self-manage their condition. Since diet may potentially affect OA, we reviewed the literature on the relationship between nutrition and OA risk or progression, aiming to provide guidance for clinicians. For overweight/obese patients, weight reduction, ideally incorporating exercise, is paramount. The association between metabolic syndrome, type-2 diabetes and OA risk or progression may partly explain the apparent benefit of dietary-lipid modification resulting from increased consumption of long-chain omega-3 fatty-acids from oily fish/fish oil supplements. A strong association between OA and raised serum cholesterol together with clinical effects in statin users suggests a potential benefit of reduction of cholesterol by dietary means. Patients should ensure that they meet the recommended intakes for micronutrients such as vitamin K, which has a role in bone/cartilage mineralization. Evidence for a role of vitamin D supplementation in OA is unconvincing.

Rheumatology key messages

- Overweight and obese OA patients should implement a weight-loss strategy incorporating exercise tailored to mobility.
- Increasing consumption of long-chain *n*-3 fatty-acids (oily fish/fish oil supplements) may improve pain and function in OA patients.
- Reducing raised blood cholesterol and increasing intake of rich vitamin K sources may benefit OA.

## Introduction

OA is the most prevalent form of arthritis [1] and the fastest growing cause of disability worldwide [2]. Globally, some 18% of women and 9.6% of men aged over 60 years have symptomatic OA, with a quarter of these individuals unable to perform routine daily activities [3]. By 2050, a projected 130 million people will suffer with OA, constituting a significant societal burden [4].

OA pathology is multifactorial, involving the remodelling of subchondral bone, synovial inflammation and loss of articular cartilage [5]. Inflammatory cytokines, notably IL-1β and TNF-α, drive catabolic pathways and perpetuate disease progression [6]. Obesity is a modifiable risk factor for OA [7] at least partly due to its associated inflammation [8]. It has recently been suggested that obesity, diabetes and the metabolic syndrome (MetS) can directly influence the development of OA [9]. A metabolic OA phenotype has been identified, which is driven by adipokines, hyperglycaemia and endocrine imbalance [1].

Current OA treatment is limited and is largely confined to symptom management [1] or total joint replacement if joint function is severely compromised [10]. Osteoarthritis Research Society International guidelines [11] recommend exercise and weight reduction in the overweight/obese, but there may be low provision of these treatments in clinical practice [2].

There is a call for a shift towards helping OA patients to self-manage their condition [11]. Since diet is a factor that may affect OA, we performed an up-to-date, literature review of the evidence for an effect of dietary factors in OA with the aim of summarizing current research findings. Guidance is targeted at clinicians, notably rheumatologists, general practitioners and dietitians and our findings form the basis of a patient information sheet (supplementary Fig. S1, available at *Rheumatology* online, and at https://www.bda.uk.com/foodfacts/OsteoArthritis.pdf).

## Methods

For this narrative review, the PubMed database was searched for articles on the effect of OA, obesity, polyunsaturated fatty acids, cholesterol and vitamins A, C, D, E and K on risk or progression of OA. The focus was on foods or nutrients that are part of the normal diet, not on nutraceuticals, which are generally consumed in pharmacological, rather than dietary, doses. Using key search terms (Table 1), searches were conducted between October 2015 and May 2017, for papers from the past 10 years, but were extended back to 2000 for vitamins A, C, D and E and to 1995 for vitamin K owing to the paucity of information available. Human studies, including observational and cohort studies, and randomized controlled trials (RCTs) were included. Excluding duplicates, 1190 articles were retrieved.

**Table 1:** Search terms used for article selection

Nutrient	Search terms
Vitamin K	Vitamin K/phylloquinone/menaquinone AND Osteoarthritis
Vitamin D	Osteoarthritis AND Vitamin D
Vitamins A, C and E	Osteoarthritis AND: Vitamin E/tocopherols/tocotrienols
	Osteoarthritis AND Vitamin C/ascorbic acid
	Osteoarthritis AND Vitamin A/carotenoids/retinol
Obesity	Osteoarthritis AND Obesity AND Progression
	Osteoarthritis AND Obesity AND Symptoms AND Review
	Osteoarthritis AND Obesity AND Risk AND Review
Polyunsaturated fatty acids	Osteoarthritis AND PUFA/polyunsaturated fatty acids/fish oil/omega-3*/*omega-three/*n*-3 fatty acids *n*-6 fatty acids
Cholesterol	Osteoarthritis AND Cholesterol/hypercholesterolaemia

Articles were examined and filtered by relevance. Papers were excluded if outcome measures could not be related to disease progression or symptoms, or quality was demonstrably poor. Sixty-eight articles were included in the final review. Where appropriate, reference lists were consulted for highly regarded older papers and primary evidence. Relevant conclusions or results were extracted from each article. Bradford–Hill criteria [12] and the critical appraisal skills programme (CASP) tool [13, 14] were used to appraise the evidence for each topic. With the heterogeneity of data rendering a systematic approach challenging, evidence is presented as a narrative review.

## Results and discussion

### Obesity

#### Consequences of obesity in OA

Obesity increases strain on weight-bearing joints [15] and, longitudinally, overweight and obese individuals are at considerably higher risk for knee arthroplasty [16]. An association of higher BMI with the development of hand OA [17] demonstrates an additional non-biomechanical role of obesity in OA. In the large Netherlands Epidemiology of Obesity cohort study, fat percentage was associated with hand OA: OR (95% CI): 1.34 (1.11, 1.61) in men and 1.26 (1.05, 1.51) in women [18]. Fat mass and waist-to-hip ratio were associated with hand OA, with an association with visceral fat in men [18]. Adiposity relates to risk of arthroplasty in knee and hip OA, with a relationship to central adiposity in knee OA [19].

**Fig. 1:**
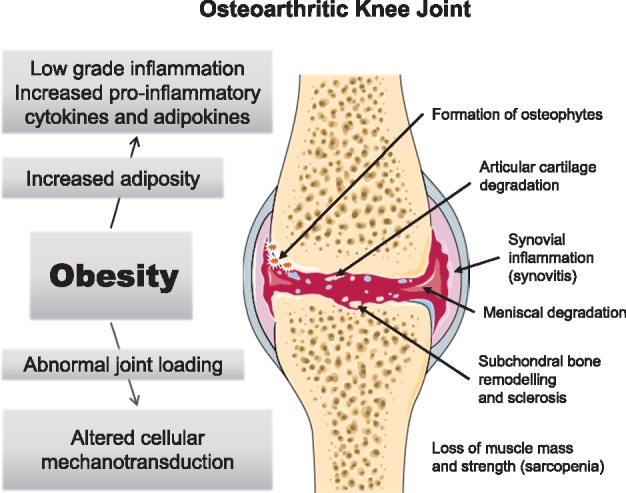
Mechanisms by which obesity leads to or exacerbates OA Adapted by permission from Macmillan Publishers Ltd. *Nat Rev Rheumatol*, Wluka AE, Lombard CB, Cicuttini FM. Tackling obesity in knee osteoarthritis [25]. Copyright 2013.

Obesity leads to low-grade systemic inflammation and weight reduction can reduce adipose tissue and restore normal secretion patterns [20]. Adipokines could comprise a critical link between obesity and OA [21]. Leptin, an adipokine generally elevated in obesity and produced by white adipose tissue of the infra-patellar fat pad, may underlie this relationship [22]. Leptin is associated with inflammation and cartilage degradation [23] and may be involved in OA pathophysiology at a local and systemic level [24]. Figure 1 summarizes the mechanisms linking obesity to OA pathogenesis [25].

##### Relationship with MetS

Central obesity, glucose intolerance (insulin resistance), dyslipidaemia and hypertension embody the MetS [26]. OA has been suggested to be a fifth component with shared mechanisms of inflammation and oxidative stress [27]. The Research on Osteoarthritis/Osteoporosis Against Disability cohort (*n* = 1384) demonstrated increased radiographic incidence and progression of knee OA with the accumulation of MetS components: ⩾3 components, for incidence, OR (95% CI): 9.83 (3.57, 27.1), *P <* 0.001; for progression: 2.80 (1.68, 4.68), *P *< 0.001 [9]. A similar effect of MetS components on knee-OA risk was seen in the Melbourne Collaborative Cohort Study though no statistically significant associations were observed for hip OA, suggesting that the pathogenesis of knee and hip OA differ [28]. Prevalence of hand OA was associated with the MetS in the Netherlands Epidemiology of Obesity cohort study in 6673 participants; adjusted OR (95% CI): 1.46 (1.06, 2.02) [29].

#### Relationship with type-2 diabetes

Hyperglycaemia prompts local accumulation of advanced-glycation end products, impairing subchondral bone and chondrocyte function [27]. In a German cohort study, type-2 diabetes was identified as an independent risk factor for severe OA and a predictor for knee/hip replacement [30]. In a French study of patients with knee OA assessed over 3 years, type-2 diabetes was a significant predictor of joint-space-width reduction in men [31].

#### Clinical effects of weight reduction on OA

As outlined in Table 2 [32–35], a systematic review and meta-analysis of RCTs revealed significant improvement in physical disability in knee OA when body-weight reduction was over 5% [35]. Subsequent clinical trials demonstrated that weight reduction reduced pain and improved function in knee OA [33, 34]. In obese participants undertaking a weight-control intervention, weight reduction reduced peak knee load and pain [32], and provided symptomatic relief, irrespective of structural damage [36].

**Table 2:** Findings of large trials and meta-analyses of weight reduction interventions in overweight/obese individuals with knee OA

Study	Participants	Intervention	Mean weight reduction from baseline	Symptom outcome change from baseline
Aaboe *et al.* [32] The CAROT Trial [33]^a^	*n* = 177 BMI: ≥30 kg/m^2^ (mean 36.9 kg/m^2^)	16 weeks, energy restricted diet (VLED/LED groups) No exercise programme; weekly contact with a dietitian	13.2% −13.7 kg (95% CI: −12.9 to −14.4; *P* < 0.0001) VLED: 12.94% ± 0.59 LED: 11.96% ± 0.55 71% lost ≥10%	30% reduction in VAS pain Mean difference: −13 mm (95% CI: −10 to −16 mm; *P* < 0.0001) 4% increase in self-selected walking speed Mean difference: 0.04 (95% CI: 0.02, 0.07) m/s; *P* < 0.0001
Messier *et al.* [34] IDEA Trial^b^	*n* = 454 BMI: ≥27 kg/m^2^ (mean 33.6 kg/m^2^)	18 months, energy restricted diet (*D + E/D/E* groups) 60 min exercise 3 days a week Contact with a dietitian weekly for the first 6 months, biweekly for the last 6 months	D + E: 11.4% −10.6 kg (95% CI: −14.1 to −7.1) D: 9.5%, E: 2% Both groups D and D + E lost significantly more weight than E (*P* < 0.001)	For D + E: 45% reduction in WOMAC pain 10% increase in walking speed and 15% increase in 6-min walk distance D + E group had significantly less pain and better function than both D and E groups
Christensen *et al.* [35] Meta-analysis of four RCTs	*n* = 454 obese patients *n* included in pooled ES: 417	6 weeks to 18 months LED/nutrition classes/meal replacements No additional exercise compared to controls. Cognitive behavioural therapy in three of the studies	6.1 (95% CI: 4.7, 7.6) kg; *P* < 0.001	Pooled ES (Outcome Measures for Arthritis Clinical Trials III): Pain: 0.20 (95% CI: 0, 0.39; *P* = 0.05) Physical disability: 0.23 (95% CI: 0.04, 0.42; *P* = 0.02) Disability could be significantly improved with >5.1% weight reduction

a The influence of weight reduction or exercise on cartilage in obese knee OA patients.

b Intensive diet and exercise for arthritis. D: diet; E: exercise; ES: effect size; LED: low energy dense; RCT: randomized controlled trial; VAS: visual analogue scale; VLED: very low energy dense.

Datasets from the Osteoarthritis Initiative and the Multicentre Osteoarthritis Study demonstrate a highly significant (*P *< 0.003) dose–response relationship between weight change and improvement in WOMAC pain and function [37]. A reduction in body weight of 10% is associated longitudinally with increased functional capacity and reduced pain in knee OA patients [37] and is additionally beneficial for metabolic health [28].

#### Physical activity as an adjunct to weight reduction

Reducing adipose tissue while maintaining muscle mass is advantageous in OA, particularly for mobility [38, 39]. Physical activity generates changes in white adipose tissue, including increased mitochondrial biogenesis and an altered adipokine profile [40], and hence weight reduction programmes that combine diet and exercise have the most benefit on functional status, joint imaging and visual analogue scale pain [41]. Meta-analysis indicates that interventions combining strengthening, flexibility and aerobic exercise are most likely to improve pain and function [42]. However, caloric restriction is still integral, as highlighted in a trial in which knee compressive forces were lowest in a diet group and inflammation (IL-6) was lowest in a diet-and-exercise group, compared with exercise alone [34] (Table 2).

#### Effective weight management

Weight reduction and maintenance of an optimal weight is challenging, particularly when mobility is impaired [25]. Activity should be tailored to the mobility, co-morbidities and preferences of the individual [43]. Successful weight reduction interventions may incorporate dietitian input [32, 36]. In clinical practice, behaviour-change strategies, increased contact and follow-up will improve adherence to weight-management programmes [44].

#### Conclusions on obesity and weight reduction in OA

Weight reduction in overweight or obese OA patients reduces joint impact and injurious loading, improves inflammatory adipokine secretion patterns and positively affects metabolic-risk profile. Effective dietary interventions do not deviate greatly from general weight-management guidelines [45]. Guidance is given in Table 3 [46–56].

**Table 3:** Summary of dietary interventions that may be of benefit in OA

Intervention	Detail of recommended interventions	Points to note
Weight reduction in overweight or obese patients	An initial aim of 10% body weight reduction should be included in a first-line approach for obese patients with OA. Overall aim for obese/overweight patients is for BMI within the healthy range (18.5–25 kg/m^2^) Dietary modification should include moderate energy restriction without compromising micronutrient intake Exercise should be encouraged including aspects of aerobic exercise, strengthening and flexibility that should be tailored to mobility	Regular clinical contact and monitoring, including dietetic input, are essential for dietary modification. Clinical input should incorporate a focus on behaviour change
Beneficial dietary-lipid modification in OA patients	Reduce intake of *n*-6 fatty acids by substituting oils rich in mono-unsaturates such as rapeseed, canola and olive oils. Aim to increase intake of long-chain *n*-3 fatty acids via a direct source of EPA/DHA; increase intake of oily fish; aim to consume a minimum one portion per week (as in general healthy eating guidelines) and preferably two Consider a daily standard fish oil supplement (1–2 capsules/day)	Women who are pregnant or breastfeeding should avoid fish with high levels of mercury (i.e. shark, swordfish and king mackerel) [46] and should avoid cod-liver oil due to the vitamin A content [47]
Dietary management of cholesterol, serum lipids and comorbidities, CVD and MetS	A cholesterol-lowering dietary portfolio should be advocated to patients with raised serum cholesterol (>5 mmol/l/>200 mg/dl) or LDL-C (>3 mmol/l/>100 mg/dl)^a^ to reduce CHD risk with the potential for OA benefit ≥2 g/day plant stanols/sterols [49] Reduce SFA intake to < 11% total energy (around 31 g/day for males and 24 g/day for females) Ensure daily intake of viscous fibre (e.g. oats), soy protein (25 g) and nuts (30 g) For obese/overweight patients, weight reduction^b^ remains of primary importance both for OA symptom management and reduction in risk of the co-morbidities, CVD and MetS	Sources of soy protein include soy milk (7.5 g soy protein per 250 ml serving), soy/edamame beans and tofu
To achieve adequate levels of vitamins A, C and E	Ensure adequate daily intake through consumption of rich dietary sources (see supplementary Table S2, available at *Rheumatology* online) Adult recommended intakes are shown below: Vitamin A (retinol equivalent): 650–750 µg/day (Europe [50]); 700–900 µg/day (USA [51])^c^ Vitamin C: 95–110 mg/day (Europe [52]); 75–90 mg (USA [51])^c^ Vitamin E (α-tocopherol equivalent): an adequate intake level of 11–13 mg/day (Europe [53]); 15 mg/day (USA [51, 107]) Only consider a multivitamin supplement if dietary intake of these nutrients is insufficient to meet dietary recommendations. Obtaining intake through diet is preferable	US guidelines suggest an additional 30 mg/day Vitamin C for smokers
To increase vitamin D intake/status	Increase consumption of vitamin-D-rich foods, for example, oily fish, eggs (yolks), vitamin-D-fortified spreads, fortified milk, fortified cereals (see supplementary Table S2, available at *Rheumatology* online) During the summer months, daily sunlight exposure (without protective cream/lotion) of approximately 10–20 min (depending on skin type, time of day, altitude and latitude) should be sufficient to produce adequate vitamin D [54, 55] With minimal sun exposure, supplementation of 15–20 µg/day should be encouraged, based on European and American guidelines, to ensure sufficient vitamin D concentration [54, 55] Maintaining a healthy BMI, that is, between 18.5 and 25 kg/m^2^, will reduce the risk of vitamin D sequestration in adipose tissue [54, 55, 126, 127]	
To increase vitamin K intake	Increase green-vegetable consumption, particularly of rich sources such as spinach, Brussels sprouts, kale and broccoli [56] (see supplementary Table S2, available at *Rheumatology* online for list of sources) Certain fats and oils (e.g. blended vegetable oil, olive oil and margarine [56]) contain small amounts of vitamin K and therefore utilizing these in cooking or as plant spreads may increase intake	The addition of a fat (such as olive oil) to a vitamin K source may increase bioavailability, as vitamin K is fat-soluble

^a^NHS reference ranges/NHLBI [48] reference ranges, ^b^See recommendations for weight-reduction in overweight/obese OA patients, and ^c^Excluding pregnant/lactating women. CVD: cardiovascular disease; DHA: docosahexaenoic acid; EPA: eicosapentaenoic acid; MetS: metabolic syndrome.

### Polyunsaturated fatty acids

#### Dietary-lipid modification in OA

Lipids are stored in the matrix and chondrocytes of articular cartilage and may contribute towards inflammation, cartilage degradation and impaired chondrocyte structure [57]. OA joints accumulate high levels of omega-6 (*n*-6) fatty acids, precursors of pro-inflammatory eicosanoids [58]. In individuals with, or at high risk of, knee OA, a positive association was seen between the *n*-6 polyunsaturated fatty acid (PUFA) arachidonic acid (AA) and synovitis but an inverse relationship between total plasma *n*-3 PUFA, docosahexaenoic acid (DHA) and patellofemoral cartilage loss, as measured by MRI [59]. With diet influencing systemic lipid levels [59], it is plausible that dietary manipulation could affect articular-cartilage composition and structural damage in knee OA. A large prospective study in OA patients found that higher intakes of total and saturated fat were associated with increased knee joint space-width loss, whereas higher intakes of monounsaturated fatty acids (MUFAs) and PUFAs were associated with reduced radiographic progression [60].

#### Rationale for an effect of PUFAs in inflammation in OA

**Fig. 2:**
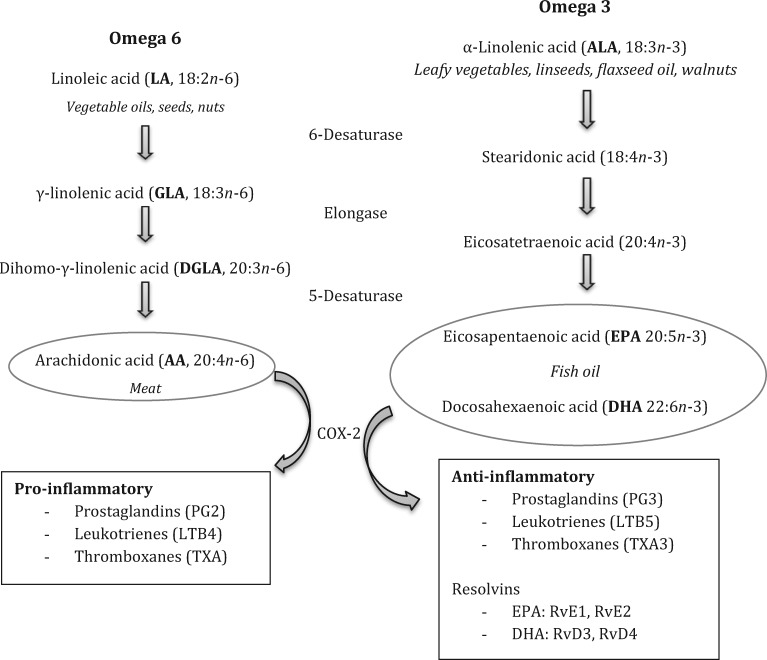
Essential fatty acids: elongation and chain saturation, dietary sources and inflammatory effects Adapted from Rayman and Callaghan [61, 62].

Eicosanoids are hormone-like agents that mediate and regulate inflammation. Figure 2 illustrates their formation from 20-carbon precursors [61, 62]. Eicosapentaenoic acid (EPA) and DHA create less potent inflammatory eicosanoids than those formed from the *n*-6 series. Indirectly, long-chain (LC) *n*-3 PUFAs decrease production of pro-inflammatory eicosanoids, reactive oxygen and nitrogen species and cytokines, additionally generating anti-inflammatory mediators (resolvins) [62].

*In vitro* models have suggested benefits of LC *n*-3 PUFA supplementation for inflamed joints, with EPA proving the most effective [63, 64]. In canine OA, fish oil *n*-3 fatty acids improved weight bearing [65] and the arthritic condition [66].

#### PUFAs in the diet

The Western diet has a high ratio of *n*-6 to *n*-3 fatty acids [67], predisposing to inflammation [59]. Within the Melbourne Collaborative Cohort Study of healthy individuals, increased *n*-6 fatty acid consumption was associated with the development of bone-marrow lesions [68]. AA originates from meat and from the conversion of linoleic acid (LA), commonly found in vegetable oils (Fig. 2). Oils high in *n*-6 fatty acids include safflower oil (79%) and sunflower oil (62.2%), with low levels in olive oil, which is rich in monounsaturates [69]. The richest source of α-linolenic acid is flaxseed oil (57%) [59], but its conversion into EPA/DHA is inefficient [70]. Increasing LC *n*-3 PUFA status to promote an anti-inflammatory effect is best achieved with direct EPA intake alongside decreased LA intake.

EPA and DHA are found primarily in oily fish [71] (see supplementary Table S1, available at *Rheumatology* online). UK intakes of oily fish are below the recommended level of ‘at least one portion/week’ [72, 73] and a significant number of US adults are not meeting American Dietary Guidelines of an average consumption of 250 mg LC *n*-3 fatty acids per day [74]. Higher intakes of EPA/DHA, including the proposed anti-inflammatory threshold of >2.7 g/day [75], may be more easily achieved by fish oil supplementation. In a large Australian study, 32.6% of the population had recently taken fish oil or *n*-3 supplements, with OA patients more likely to use them [76].

#### EPA/DHA supplementation

The use of fish oils has shown clinical efficacy for pain reduction in RA [61, 77], but published trials of LC *n*-3 fatty acid supplementation in OA are limited. A systematic review and meta-analysis concluded that there was no statistically significant effect of marine oil supplements for OA pain (five trials; −0.17; 95% CI: −0.57, 0.24) [78]. However the quality of the trials included was graded as ‘very low’, and the results for the OA group were highly heterogeneous leading the authors to state that the evidence was not sufficiently robust to determine the effect of marine oil in patients with diagnoses other than RA [78]. Hill and colleagues [79] conducted a double-blind 2-year RCT in 202 knee-OA patients. There was no placebo group, attributed to efforts to maintain blinding and for ethical reasons [79]. Unexpectedly, greater benefits to WOMAC pain and function scores were found from a low-dose (0.45 g LC *n*-3) fish oil supplement than from a high, anti-inflammatory dose (4.5 g LC *n*-3) [79]. The authors concluded that there was no additional benefit of high-dose over low-dose fish oil in knee OA [79]. The low-dose supplement comparator was composed largely of Sunola oil, a monounsaturated *n*-9 fatty acid. Unlike olive oil, it is not rich in polyphenols with known antioxidant and potentially anti-inflammatory effects [80]. However, there may have been benefit to pain from the high oleic acid content [81], which may have effectively constituted a second active intervention [82].

#### Conclusions on importance of PUFAs in OA

Adding complexity to supplementation trials, the effects of fish oil can be affected by polymorphisms that alter the expression of cytokine genes [62] and it is difficult to find an appropriate placebo oil. Though further studies with appropriate controls are needed, there may be efficacy for pain reduction with a low-dose supplement equivalent to 1.5 standard capsules of 1 g fish oil/day [79]. Moreover, there is evidence for fish oil being of benefit to cardiovascular health [83], which may be relevant to this population owing to the association of OA with MetS [27]. Recommendations are summarized in Table 3.

### Cholesterol

#### Links between elevated blood cholesterol and OA

Epidemiological studies have implicated serum cholesterol as a systemic OA risk factor [84–86]. In women from the Chingford study, knee OA was significantly associated with moderately raised serum cholesterol (OR = 2.06; 95% CI: 1.06, 3.98) [84]. In a cross-sectional study of patients with OA-related arthroplasty, hypercholesterolaemia, defined as ⩾6.2 mmol/l (239 mg/dl), was independently associated with generalized OA [85]. Hand OA has recently been associated with documented hypercholesterolaemia [86]. Longitudinal data have strengthened the evidence; in an Australian cohort of healthy women, for every 1 mmol/l increase in total cholesterol, the adjusted odds of developing a bone-marrow lesion were 1.84 (95% CI: 1.01, 3.36), *P *= 0.048 [87].

#### Cholesterol accumulation in OA

Cellular cholesterol accumulation is known to induce cytotoxicity [88] and hypercholesterolaemia can increase AA formation and production of pro-inflammatory eicosanoids [89]. Cholesterol has been found to accumulate in human OA cartilage [90]. Accumulation is typically prevented through the cholesterol efflux system [88], which may be dysregulated in OA [91]. An association has been found between OA pathogenesis and a single nucleotide polymorphism in *SRBP-2*, the gene for a protein involved in cholesterol homeostasis [92]. Furthermore, low-density lipoprotein (LDL)-cholesterol appears to influence OA development and progression [93]. In mice induced with OA and fed a cholesterol-rich diet, LDL accumulation led to increased synovial activation and osteophyte formation [94]. Oxidized LDL, in particular, may be involved in synovial inflammation and cartilage destruction [93].

#### Does reducing serum lipids reduce the burden of OA?

Reducing cholesterol accumulation with statins appears to have favourable effects in OA. A 10-year longitudinal study in a large cohort (*n *= 16 609) found that an increasing therapeutic statin dose *vs* no statin dose was incrementally associated with a decreased incidence of clinical OA [95]. In a further cohort, statin use was associated with over 50% reduction in radiographic progression of knee OA [OR (95% CI): 0.43 (0.25, 0.77)] [96]. Statins also reduce the expression of inflammatory cytokines and attenuate inflammation in OA [97]; they have been shown to exert chondroprotective effects by reducing cartilage degradation, with atorvastatin treatment inhibiting IL-1β and expression of MMP-13 *in vitro* [98].

#### Cholesterol-lowering dietary strategies applicable to OA

**Fig. 3:**
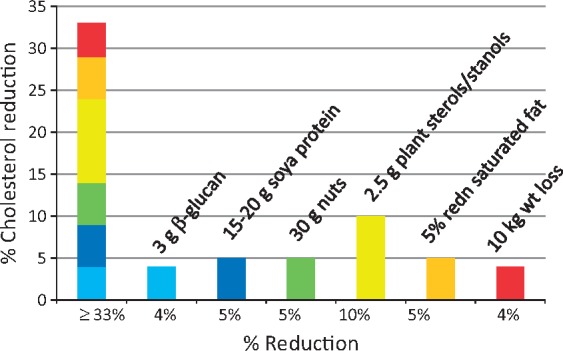
Dietary potential to lower cholesterol: different dietary strategies can add up to a total cholesterol reduction of >30% Information for this figure was taken from [98, 99].

Cholesterol can also be lowered by dietary strategies [99]. Assuming additive effects, dietary changes could result in a 35% reduction in LDL-cholesterol, equivalent to that of a starting dose of statins [99, 100] (Fig. 3). Energy restricted weight reduction is of primary importance for reducing LDL-cholesterol in the overweight and obese [99, 101]. Saturated fat should be reduced to 11% of total energy with PUFAs and MUFAs acting as favourable substitutes [100]. Dietary cholesterol (as in eggs) is now known to exert a relatively insignificant effect on serum cholesterol compared with that of saturated fatty acids [100]. Viscous (soluble) fibre appears to lower serum cholesterol by 5–10% for a 5–10 g daily dose [102] and oat (β)-glucans have been found to be effective in a 3 g dose [103]. Meta-analysis concluded that 25 g soy protein/day contributed to total and LDL-cholesterol reduction [104]. There is strong evidence that plant stanols and sterols lower LDL-cholesterol [49]. Their richest source is fortified proprietary spreads, drinks and yogurts. A meta-analysis of 41 trials showed that intake of 2 g/day of stanols/sterols reduced LDL-cholesterol by 10% [49]. There appears to be a dose–effect relationship such that a dose of 9–10 g of plant stanols lowered LDL-cholesterol by 18% [105]. Nuts provide a source of unsaturated fats, soluble fibre and phytosterols [106]. A meta-analysis of 61 intervention trials found that tree nuts (e.g. almonds, walnuts and pistachios) significantly lowered total and LDL-cholesterol [107]. Including 30 g/day [108] of nuts in the diet could provide a simple cholesterol-lowering strategy. Dietary interventions to lower cholesterol and serum lipids and manage comorbidities are given in Table 3.

### Antioxidants and OA

A plausible rationale exists for a role of antioxidants in OA; reactive oxygen species and reactive nitrogen species may be involved in the pathophysiology of OA, and therefore suppressing these with antioxidants might delay its onset and progression [109, 110]. The antioxidant vitamins, A, C and E have received the most attention in this context with vitamin C being particularly relevant owing to its requirement for collagen formation [111].

#### Human studies on antioxidants and OA

Trials so far have primarily focused on the effects of vitamin E in OA [112, 113]. A systematic review of trials has investigated all three nutrients, although seven of the nine studies included were vitamin E trials [113]. Findings were conflicting, with only two short-term trials suggesting a beneficial effect of vitamin E [113]. Though vitamin C supplementation appeared to reduce OA pain, that effect has not been reproduced in other trials. A later trial of vitamin E supplementation (200 mg/day) in knee OA found a significant benefit on pain and significantly increased levels of circulating antioxidant enzymes [112]. No study to date has solely investigated vitamin A supplementation in OA, though when combined (as β-carotene) with vitamins C, E and selenium, no effect was seen [113]. Unfortunately, most of these studies were of poor quality with variations in duration, sample size, measurement outcomes, supplement dosage and form [112, 113].

#### Conclusions on antioxidant vitamins

At present, there are insufficient data to show benefit from antioxidant supplementation in OA. However, for general health, patients should be encouraged to eat a healthy diet that includes adequate amounts of dietary antioxidants (see recommendations in Table 3 [50–53]).

### Vitamin D and OA

Although vitamin D has many biological roles, its primary function is thought to be the regulation of bone metabolism and calcium homeostasis [114]. The majority of its activity occurs via the vitamin D receptors (VDRs), a subfamily of nuclear receptors that regulate gene expression, to which it binds with high affinity [114].

#### Postulated role of vitamin D in OA

Acting through the VDR, vitamin D has a major role in the regulation of mineral homeostasis and bone metabolism [114]. Thus, inadequate vitamin D status is thought to impair the ability of bone to respond to the pathophysiological process of OA and influence disease progression [114, 115]. Vitamin D is also believed to have effects on inflammation and cytokine synthesis [115]. Furthermore, a number of trials have shown that vitamin D supplementation has positive effects on muscle strength [116, 117]; this may be beneficial in OA, which is often associated with marked weakness of the quadriceps muscles [118].

#### Human evidence on vitamin D in OA

Results from observational studies, predominantly in knee OA, though inconsistent, suggest a positive association between vitamin D deficiency, cartilage loss and OA prevalence/progression [119, 120].

Trials have had mixed findings. Three RCTs found no significant effect of vitamin D supplementation on cartilage volume or pain in knee OA [121–123], though in a *post hoc* analysis of one study, significant improvements in total WOMAC score and WOMAC function were found [122]. Only one RCT has had positive results, viz., a significant decrease in OA pain, and an increase in symptomatic knee function [124]. A small non-controlled intervention also found a significant increase in muscle strength following two months of vitamin D supplementation [125]. In a recent trial, people with knee OA who consistently maintained sufficient plasma vitamin D (>50 nmol/l) had significantly improved structural and functional outcomes than those consistently insufficient, suggesting a level of vitamin D status to aim for [126].

#### Alternative explanation for role of vitamin D in OA

There are alternative theories as to why vitamin D supplementation has not translated into positive outcomes [127, 128]. Emerging evidence suggests that low vitamin D may simply be a marker of ill-health across a number of conditions [127], for example, the inflammatory process and clinical course of OA could result in vitamin D deficiency as a consequence, rather than a cause, of disease [127]. Indeed, low serum vitamin D in inflammatory conditions may be due to the dysregulation of the VDR [128].

#### Conclusions on vitamin D intake/status and OA

Though it appears unlikely that vitamin D deficiency is a causal factor in OA, there appear to be benefits for muscle strength [116, 117] when patients maintain adequate vitamin D status. Vitamin D deficiency is widespread across the world [129]. Although both the Institute of Medicine [54] and the European Food Safety Authority (EFSA) [55] consider that a dietary intake that achieves a serum 25(OH)D concentration of 50 nmol/l is sufficient, other organizations prefer to define sufficiency as the higher value of 75 nmol/l [130]. Clinicians should measure vitamin D status in their OA patients and use the information in Table 3 [54, 55] to ensure that their vitamin D status reaches at least 50 nmol/l.

### Vitamin K and OA

Vitamin K is a group of fat-soluble compounds, with two naturally occurring forms, vitamin K1 (phylloquinones) and vitamin K2 (menaquinones) [131]. Vitamin K1, synthesized by plants and algae, is the form most widely found in the human diet, mainly in green leafy vegetables and oils [56]. Vitamin K2 is predominantly produced by bacteria [30].

#### Postulated role of vitamin K in OA

Aside from its role in the complement cascade [131], vitamin K is involved in bone and cartilage mineralization [132, 133]; it is a cofactor for the enzyme γ-glutamyl carboxylase, which is responsible for the γ-carboxylation and functionality of vitamin-K-dependent (VKD) proteins [132, 133]. VKD proteins found in bone and cartilage include matrix Gla protein, periostin, gla-rich protein, gas 6 and osteocalcin. Inadequate vitamin K intake may lead to decreased carboxylation of these VKD proteins, affecting functional status [132] and resulting in abnormalities that parallel those seen in OA [133].

#### Human evidence on vitamin K in OA

Studies addressing the effects of vitamin K on OA *in vivo* are very limited and most have investigated only phylloquinone. Cross-sectional studies have shown a positive association between concentrations of plasma phylloquinone <1.0 nM and the development of OA [134] and an inverse association between knee OA and plasma phylloquinone [135]. Furthermore, phylloquinone intake has been inversely associated with individual and multiple inflammatory markers [136]. In longitudinal studies, vitamin K-deficient subjects were found to be more likely to have articular cartilage and meniscus damage, often developing OA in one or both knees [137, 138].

Despite these promising results, the only randomized, controlled trial of vitamin K failed to find a significant beneficial effect of phylloquinone supplementation against hand-OA progression [139]. However, only a subsection of the group was vitamin K insufficient at baseline and, in that subset, those who attained sufficient concentrations at follow-up had 47% less joint space narrowing (*P* = 0.02).

#### Conclusions on vitamin K and OA

As the majority of epidemiological evidence thus far is observational, causality cannot be demonstrated; however, the essential role of vitamin K in bone and cartilage health is incontrovertible. Not all populations reach the recommended vitamin K intake. Furthermore, there is no gold-standard biomarker [140]. While studies from Germany, America, the Netherlands, Japan and parts of Northern China have shown adequate intake of vitamin K according to guidelines [141–145], 59% of UK adults >65 years had an intake below recommendations, probably because of inadequate consumption of cooked green vegetables [146]. Guidance to increase vitamin K intake is given in Table 3 [56].

## Conclusions

Despite the limitations of evidence that is largely based on observational studies, most of which are on knee OA, this review can offer some guidance to clinicians. With excess adiposity appearing to underlie the metabolic factors now recognized as being integral to OA, particularly of the hand and knee [28, 29], dietary modification to achieve weight reduction where appropriate, together with increased physical activity, are the strongest evidence-based recommendations. Though the evidence for benefit of dietary-lipid modification (increased LC *n*-3 PUFA/decreased LC *n*-6 PUFA) and lowering of serum cholesterol on OA is currently somewhat sparse, the recommendations proposed will at least benefit metabolic health. While vitamin/micronutrient data are limited, there is a plausible role for these nutrients in preventing/slowing OA, though at what intake levels remains to be seen. The accompanying patient-information sheet (supplementary Fig. 1, available at *Rheumatology* online; https://www.bda.uk.com/foodfacts/OsteoArthritis.pdf) summarizes potentially beneficial recommendations and is designed for healthcare professionals to distribute to their patients.

An ageing population and the current obesity epidemic [147] predict an increased global burden of OA. Given the current paucity of treatment options, any risk-free means of reducing progression or relieving debilitating symptoms in such a large patient group should be given a try. While we await well-designed, larger scale RCTs in OA patients of clearly defined phenotype, preferably relatively early in the disease process, our findings could be used to support the development of guidelines using the Delphi process of expert consensus to make recommendations.

## Supplementary Material

Supplementary Data
