# Supplementary material for: What is the evidence for a role for diet and nutrition in osteoarthritis?
Source: Rheumatology (Oxford). 2018 Apr 17;57(Suppl 4):iv61–74. doi: 10.1093/rheumatology/key011 (PMC5905611; doi:10.1093/rheumatology/key011)
Supplement: Supplementary Data [file key011_rhe-17-0209-file005.docx]

**SUPPLEMENTARY DATA**

**Supplementary figure S1. Food Fact Sheet: Diet and osteoarthritis**
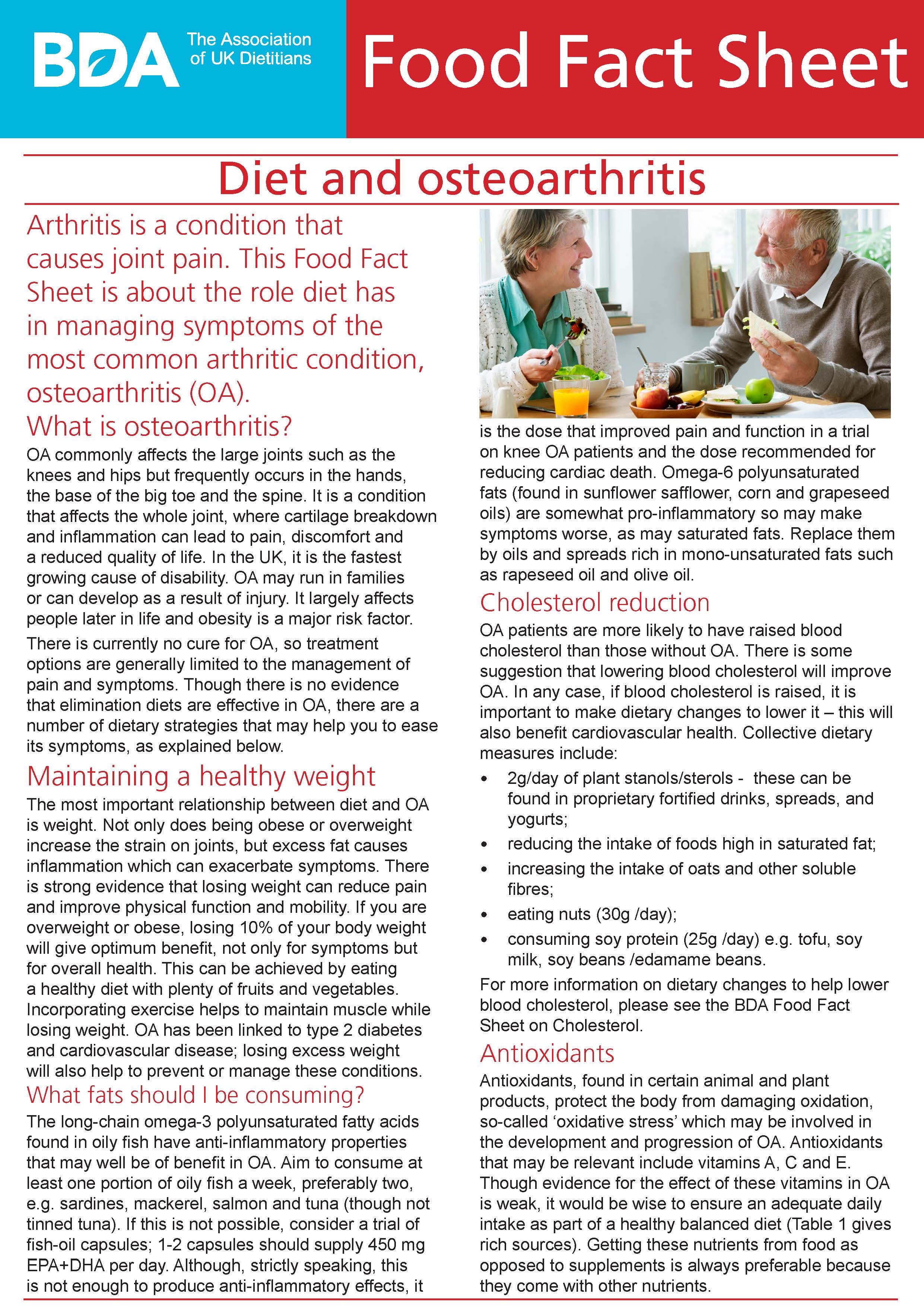


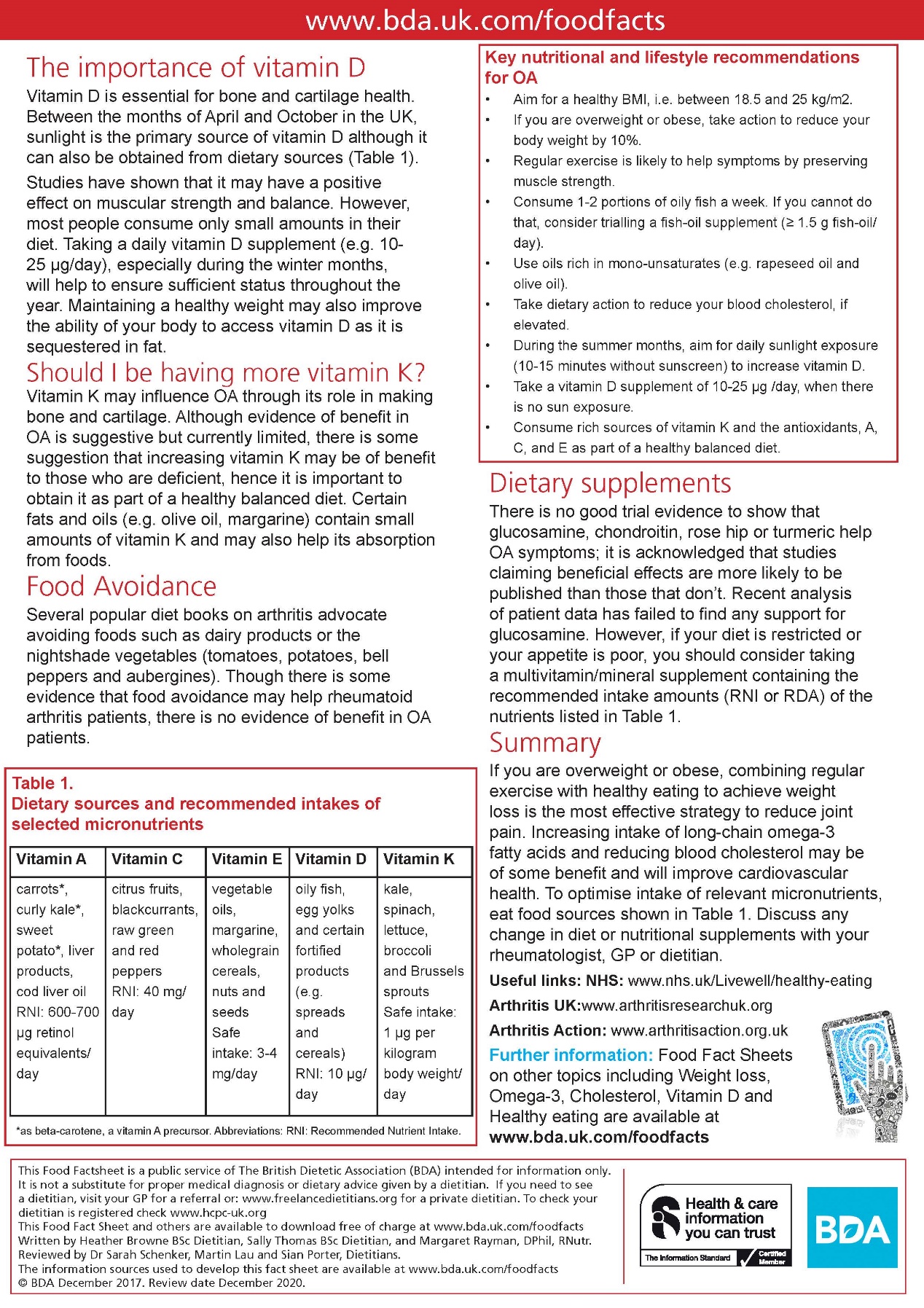


**Supplementary Table S1.** **Typical long chain n-3 fatty acid content of fish and seafood per 100g food**

| **Food** | **Total LC n-3 per 100 g food, g^a^** | **EPA per 100 g food, g^b^** | **DHA per 100 g food, g^c^** |
| --- | --- | --- | --- |
| Cod | 0.07 | 0.02 | 0.05 |
| Cod liver oil | 20.5 | 10.80 | 8.30 |
| Crab (white meat, cooked) | 0.06 | 0.04 | 0.02 |
| Haddock | 0.08 | 0.02 | 0.06 |
| Herring | 1.31 | 0.51 | 0.69 |
| Kippers (grilled) | 2.49 | 1.10 | 1.28 |
| Mackerel | 2.76 | 0.95 | 1.65 |
| Mussels (cooked) | 0.49 | 0.34 | 0.13 |
| Plaice | 0.16 | 0.07 | 0.09 |
| Salmon (fresh, farmed) | 2.55 | 0.77 | 1.44 |
| Salmon (fresh, wild) | 1.92 | 0.57 | 1.07 |
| Salmon (pink, canned in brine) | 1.07 | 0.35 | 0.63 |
| Sardines (fresh) | 1.19 | 0.49 | 0.62 |
| Sardines (canned in brine) | 2.17 | 1.10 | 0.92 |
| Tuna (fresh) | 0.08 | 0.01 | 0.07 |
| Tuna (canned in brine) | 0.30 | 0.04 | 0.25 |
| Tuna (canned in oil) | 0.16 | 0.02 | 0.14 |

Data for this table can be found in [1].^a^Chain length 20 carbons or more: includes 20:5 (EPA), 22:5 and 22:6 (DHA). ^b^Eicosapentaenoic acid (cis n-3 20:5). ^c^Docosahexaenoic acid (cis n-3 22:6).

**Supplementary Table S2**. **Micronutrient content of various food sources per 100g of food**

| **Vitamin D** | **Amount per 100g of food** |
| --- | --- |
| Herring | 16.1µg |
| Trout | 9.6µg |
| Mackerel | 8.8µg |
| Pilchards (tinned in tomato sauce) | 14.0µg |
| Salmon | 7.1µg |
| Sardines (tinned in tomato sauce) | 8.0µg |
| Shiitake mushrooms (sun-dried) | 41.5µg |
| Eggs | 1.8µg |
| Margarine | 7.9µg |
| Fortified breakfast cereals | 2.8-8.3µcg |
| **Vitamin A** | **Amount of retinol equivalents per 100g of food** |
| Carrots | 1850µg |
| Curly kale | 560µg |
| Sweet potato | 855µg |
| Liver products | >7300µg |
| Dairy products | 10-1000µg |
| Cod liver oil | 18000µg |
| **Vitamin C** | **Amount per 100g of food** |
| Citrus fruits | >50mg |
| Blackcurrants | 115mg |
| Raw green and red peppers | 120mg |
| Fruit Juice | 33mg |
| **Vitamin E** | **Amount per 100g of food** |
| Sunflower seeds | 37.8mg |
| Pecan nuts | 26.7mg |
| Almonds | 24mg |
| Hazelnuts | 25mg |
| Pine nuts | 11.1mg |
| Peanuts | 10.1mg |
| Brazil nuts | 14.4mg |
| Sunflower oil | 49.2mg |
| Palm oil | 33.1mg |
| Soya bean oil | 18.2mg |
| **Vitamin K** | **Amount per 100g of food** |
| Spinach | 575µg |
| Lettuce | 129µg |
| Broccoli | 131µg |
| Brussels Sprouts | 122µg |
| Blended vegetable oil | 114µg |
| Olive oil | 54.8µg |
| Margarine | 43µg |

Adapted from [1-3].

**References**

1. McCance and Widdowson’s composition of foods integrated dataset, (CoFID) - Publications - GOV.UK [Internet]. Gov.uk. 2015. Available from:<https://www.gov.uk/government/publications/composition-of-foods-integrated-dataset-cofid> (accessed 16 January 2017).
2. McLaughlin PJ, Weihrauch JL. Vitamin E content of food. [J Am Diet Assoc](https://www.ncbi.nlm.nih.gov/pubmed/389993) 1979;75(6):647-65.
3. Bolton-Smith C, Price RJ, Fenton ST, Harrington DJ, Shearer MJ. Compilation of a provisional UK database for the phylloquinone (vitamin K1) content of foods. Br J Nutr 2000;83:389–99.
